# Phylogenetic Analysis of Conservation Priorities for Aquatic Mammals and Their Terrestrial Relatives, with a Comparison of Methods

**DOI:** 10.1371/journal.pone.0022562

**Published:** 2011-07-25

**Authors:** Laura J. May-Collado, Ingi Agnarsson

**Affiliations:** 1 Department of Biology, University of Puerto Rico, San Juan, Puerto Rico; 2 Department of Environmental Sciences and Policy, George Mason University, Fairfax, Virginia, United States of America; University of York, United Kingdom

## Abstract

**Background:**

Habitat loss and overexploitation are among the primary factors threatening populations of many mammal species. Recently, aquatic mammals have been highlighted as particularly vulnerable. Here we test (1) if aquatic mammals emerge as more phylogenetically urgent conservation priorities than their terrestrial relatives, and (2) if high priority species are receiving sufficient conservation effort. We also compare results among some phylogenetic conservation methods.

**Methodology/Principal Findings:**

A phylogenetic analysis of conservation priorities for all 620 species of Cetartiodactyla and Carnivora, including most aquatic mammals. Conservation priority ranking of aquatic versus terrestrial species is approximately proportional to their diversity. However, nearly all obligated freshwater cetartiodactylans are among the top conservation priority species. Further, ∼74% and 40% of fully aquatic cetartiodactylans and carnivores, respectively, are either threatened or data deficient, more so than their terrestrial relatives. Strikingly, only 3% of all ‘high priority’ species are thought to be stable. An overwhelming 97% of these species thus either show decreasing population trends (87%) or are insufficiently known (10%). Furthermore, a disproportional number of highly evolutionarily distinct species are experiencing population decline, thus, such species should be closely monitored even if not currently threatened. Comparison among methods reveals that exact species ranking differs considerably among methods, nevertheless, most top priority species consistently rank high under any method. While we here favor one approach, we also suggest that a consensus approach may be useful when methods disagree.

**Conclusions/Significance:**

These results reinforce prior findings, suggesting there is an urgent need to gather basic conservation data for aquatic mammals, and special conservation focus is needed on those confined to freshwater. That evolutionarily distinct—and thus ‘biodiverse’—species are faring relatively poorly is alarming and requires further study. Our results offer a detailed guide to phylogeny-based conservation prioritization for these two orders.

## Introduction

The ongoing biodiversity crisis is significantly effecting mammals and between 21% and 36% of the 5,847 extant mammalian species are threatened [1]. About 76 species have gone extinct since 1500s, and an additional 29 critically endangered species are thought to be on the brink of extinction [1], [2]. Extensive human land use, global climate change, and hunting and by-catch are the main factors affecting mammalian populations worldwide, in some cases causing rapid local and regional defaunation [1], [3]. Schipper et al. [1] proposed aquatic mammals as particularly vulnerable to current threats to marine and freshwater environments including pollution, intense harvesting (e.g., of minke whales, harp seals) [4]–[7], climate change (e.g., polar bear, walrus, fur seals, and narwhals) [8]–[12] and high incidental mortality in fishing nets (e.g., small cetaceans, fur seals) [13]–[18]. In light of such threats, and faced with limited resources, establishing conservation priorities for aquatic and terrestrial mammals is an urgent task.

Many criteria are being used to prioritize conservation effort. Prominently, the IUCN Red List establishes the imperilment of species based on several criteria including population size, distribution, fragmentation, and rate of decline of populations [19]. In addition to risk, factors unique to each species may influence conservation decisions, including the ecological role of species, species “charisma”, and cost and feasibility of successful conservation [20], as well as ‘latent extinction risks” based on species biological traits [21].

Recently, the evolutionary history of species and lineages has begun to be considered as well, and such information is increasingly being used to establish conservation priorities [22]–[29]. Species differ in the amount of unique evolutionary history they represent. The loss of evolutionarily unique species with no close relatives represents a greater loss of biodiversity than the loss of a species whose evolutionary history is, to a large degree, shared with one or more closely related species. In other words, the extinction of a single species could have a minor effect on the tree of life if that species has many close relatives, while on a species-poor branch its loss could extinguish that entire branch. Therefore, phylogenies provide an additional measure of biodiversity that compliments species richness and thus considering evolutionary distinctiveness should play a role in prioritizing species for conservation, if the goal is to maximally conserve biodiversity.

A combination of criteria including both evolutionary distinctiveness and level of imperilment may thus provide a good assessment of where conservation efforts may be most urgent [30]. This prioritizing of species can be achieved by using EDGE [31] and HEDGE [26] metrics, which consider both evolutionary distinctiveness (i.e. how much unique evolutionary history the species represent) as well as extinction risk. Use of these kinds of methods underlies the EDGE program [32], a global initiative, which focuses on the conservation of ‘one-of-a-kind species’, that is, threatened species that are highly evolutionarily distinct. The EDGE program highlights the potential for these methods to be used in conservation research. Phylogenies also have revealed that extinction risk is phylogenetically non-random, implying that the biological traits of groups of closely related species (clades) affects how species respond to human impact [33], [34]. Thus phylogenies can help us understand why species are at risk and assist in the prediction of future risk of species.

To date, the most comprehensive study estimating phylogenetic conservation priorities for mammals is Isaac et al. [31]. In that landmark study, they rank species of ‘all’ mammals and thus include both orders considered here. However, (1) several Cetartiodactyla and Carnivora species were missing from their phylogeny, approximately 10% of currently recognized species, to the best of our knowledge; (2) they used a mammalian supertree with relatively low resolution; and (3) they considered only one, and arguably not the most appropriate, of the available approaches to estimate conservation priorities. In this study, we prefer one particular approach, but also consider how sensitive the results are to choice among a range of available methodologies, including the EDGE and HEDGE metrics [see 27] and propose a consensus approach that may be useful when species ranks differ among methods. Furthermore, we use virtually species-complete phylogenies for the two orders containing most of the aquatic mammal diversity (modified phylogenies of Cetartiodactyla [35] and Carnivora [36]). We estimate conservation priorities for species to provide a more detailed ranking of conservation priorities than prior studies, and specifically test (1) if aquatic mammals emerge as more urgent conservation priorities than their terrestrial relatives and (2) examine if current conservation effort for high priority species is successful.

## Materials and Methods

We use the most detailed primary-data species-level phylogenies available [35], [36]. However, these phylogenies did not include all species, hence we added the missing species to reconstruct phylogenies including all 333 Cetartiodactyla and 287 extant Carnivora taxa prior to conservation-priority analyses. To ensure we added all described species of each order to the original phylogenies we used the detailed Youtheria [37] and IUCN Red List databases [19]. ‘Missing’ taxa from [35], [36] were added using the following approaches. Species for which DNA data had just recently become available in Genbank were simply added to the matrices and analyses rerun using the same settings as in Agnarsson and May-Collado [35] and Agnarsson et al. [36]. For the remaining species we added them manually according with their placement in (1) the mammal supertree [38], and (2) for species absent in the supertree we added them according to current taxonomy. Manually added species were added unresolved at the base of their least inclusive taxonomic unit (usually genus), unless their placement was more exactly indicated in the mammal supertree. Branch lengths of manually added taxa were assumed to be approximately equal to their sister taxon when placed ‘precisely’, or represent averages of other terminal taxa in the least inclusive taxonomic group when placed as unresolved at the base of the taxon.

Extinction risk status data was obtained from The IUCN Red List of Threatened Species 2010.4 [19] and translated to a continuous index representing estimated % of risk of extinction [27], [39].

Many methods exist to integrate IUCN data with phylogenetic information to establish conservation priorities, and which approach is best is debated in e.g. Faith [40] and Mooers et al. [27]. For example, Mooers et al. [27] summarize five different methods to transform IUCN risk categories to % extinction risk. Once a transformation method has been chosen, one then has a choice among methods to establish evolutionary/phylogenetic distinctiveness. Faith [40] e.g. argues that the phylogenetic distinctiveness class of methods (PD) outperforms the ‘standard’ EDGE methodology. This is because PD methods such as HEDGE considers the extinction probabilities of relatives, when estimating the contribution of a given species to evolutionary diversity [40], [41]. Finally, one may choose to consider the ‘raw’ branch lengths of phylogenetic trees as informative as they represent unique evolutionary information contained in terminal taxa, or alternatively, focus on the relative placement of taxa on the tree by ultrametricizing the trees prior to analyses. These are but a few of the possible choices, yet result in 20 different analyses to establish conservation priorities, the variation among which has barely been explored. Here, we estimate the sensitivity of the results to a priori choice of criteria for transforming IUCN values to extinction risk, using the five translation methods discussed in Mooers et al. [27]: “Isaac”, “Pessimistic”, “IUCN 50”, “IUCN 100” and “IUCN 500”. We also use two distinct methodologies, the ‘traditional’ EDGE approach and a phylogenetic diversity [40] type method, HEDGE [see 41]. Furthermore, we ran analyses both across trees with ‘raw’ branch lengths as estimated by MrBayes, as well as using ultrametricized trees. A priori we favor one approach, namely the HEDGE analysis of the ‘pessimistic’ transformed data on the ‘raw branch length’ phylogeny. We agree with Faith [40] and Kuntner et al. [41] that HEDGE as a phylogenetic diversity (PD) type approach, better achieves the goal of phylogeny-based conservation than EDGE [see above and 41 for detail]. In addition, we prefer the “Pessimistic” transformation method over the others as it seems more realistic to assume that practically all species are at some considerable risk of extinction [36]. The other transformation methods assume that species in the ‘least concern’ category are essentially ‘safe’, being at very low % risk of extinction. However, monitoring IUCN categories over time shows that species status may often change rapidly; few species seem safe in the long run. Finally, we favor using ‘raw’ branch lengths as that approach more fully utilizes information from the tree: branches contain information about evolutionary uniqueness of terminal taxa, beyond the mere placement of species. Nevertheless, we also see merit in comparing results among methods, as arguably species that emerge as high priorities regardless of methodology are indisputably important. Thus, while we present in detail the conservation priority ranking of one among the set of methods, we also highlight the congruence among methods, measure simply as species shared among analyses in the top-30 list suggested by each method, and highlight top priority conservation species which all methods rank highly.

For species for which the Red List does not estimate extinction risk due to insufficient information (data deficient - DD), we arbitrarily assigned an extinction risk value in between the two lowest IUCN Red List categories (least concern and near threatened). This is a conservative estimate, made simply to be able to include these species in the analysis. Conservative, because presumably in many cases the reason species are too poorly known to be IUCN-listed is limited distribution and/or absolute rarity, which, if better known, might place them in higher risk categories that those here indicated.

We performed our analyses of conservation priorities integrating IUCN categories and the evolutionary history of species using the TUATARA module version 1.01 [42] in the evolutionary analysis package MESQUITE version 2.74 [43]. For each of the transformations we used two metrics: the Evolutionarily Distinct, Globally Endangered (EDGE) metric, and “heightened ” EDGE (HEDGE) metric. Both define species priority ranks given the phylogeny and the probabilities of extinction [27], with HEDGE in addition considering the probabilities of other species going extinct to calculate the ‘expected terminal branch length’ of taxa after some episodes of extinction [a PD]-[style approach, see 40, 41 for details].

Finally, to examine if evolutionarily distinct species (high ED) are facing particularly great threats we estimated ED in Mesquite. We then calculated the number of species with populations declining, unknown, stable, and on the increase, among all species of both orders, and compared that to the population status of the top 60 ED list using a chi-square test.

## Results

### Conservation Priorities based on HEDGE

**Figure 1:**
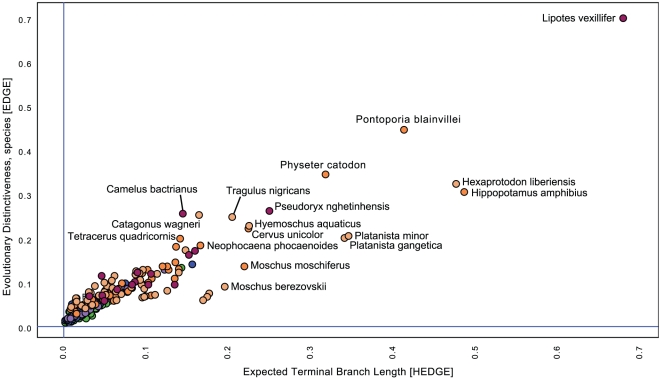
Conservation priorities based on the agreement between EDGE and HEDGE for Cetartiodactyla using the pessimistic transformation. (Red dots = Critically Endangered, Orange dots = Endangered, Yellow dots = Vulnerable, Dark Green dots = Least Concern, Light Green = Near Threat, Blue = Data Deficient).

**Figure 2:**
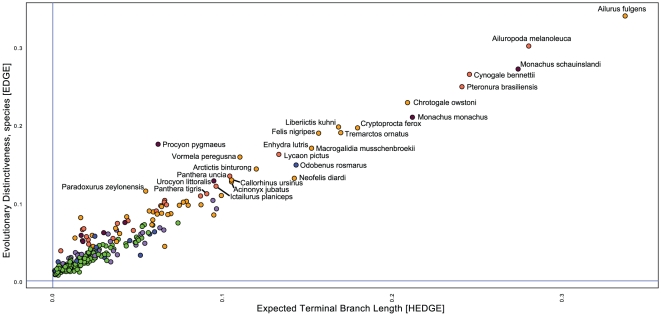
Conservation priorities based on the agreement between EDGE and HEDGE for Carnivora using the pessimistic transformation. (Red dots = Critically Endangered, Orange dots = Endangered, Yellow dots = Vulnerable, Dark Green dots = Least Concern, Light Green = Near Threat, Blue = Data Deficient).

We focus on the results of the preferred analysis, HEDGE of the ‘pessimistic’ transformed data (Figures 1,2, Tables 1, 2,3, Table S1, S2), however, in general EDGE results are similar and conservation priority species both methodologies agree on are highlighted in Figures 1 and 2 (see also Table 3, S2). The top ranking species from the ‘consensus’ approach also are, to a large degree, shared with the HEDGE-pessimistic approach (Table 3, S2).

**Table 1:** Top 30 conservation priority cetartiodactylan species according with HEDGE analysis of the ‘pessimistic’ transformed data.

Rank	Species	Common name	IUCN Extinction Risk	IUCN Population Status	IUCN System
**1**	***Lipotes vexillifer***	**Baiji**	**Critically Endangered**	**Unknown**	**F**
**2**	***Hippopotamus amphibius***	**Hippopotamus**	**Vulnerable**	**Decreasing**	**T,F**
**3**	***Hexaprotodon liberiensis***	**Pygmy Hippopotamus**	**Endangered**	**Decreasing**	**T,F**
**4**	***Pontoporia blainvillei***	**Franciscana**	**Vulnerable**	**Decreasing**	**F**
5	*Platanista minor*	Indus River Dolphin	Endangered	Decreasing	F
6	*Platanista gangetica*	Ganges River Dolphin	Endangered	Decreasing	F
**7**	***Physeter catodon***	**Sperm Whale**	**Vulnerable**	**Unknown**	**M**
**8**	***Pseudoryx nghetinhensis***	**Saola**	**Critically Endangered**	**Decreasing**	**T**
**9**	***Hyemoschus aquaticus***	Water Chevrotain	**Endangered**	**Decreasing**	**T**
**10**	***Cervus (Rusa) unicolor***	Sambar	**Vulnerable**	**Decreasing**	**T**
11	*Moschus moschiferus*	Siberian Musk Deer	Vulnerable	Decreasing	T
**12**	***Tragulus nigricans***	Balabac Maouse Deer	**Endangered**	**Decreasing**	**T**
13	*Moschus berezovskii*	Forest Musk Deer	Endangered	Decreasing	T
14	*Moschus anhuiensis*	Anhui Musk Deer	Endangered	Decreasing	T
15	*Moschus chrysogaster*	Alpine Musk Deer	Endangered	Decreasing	T
16	*Moschus fuscus*	Black Musk Deer	Endangered	Decreasing	T
17	*Moschus leucogaster*	Himalayan Musk Deer	Endangered	Decreasing	T
18	*Moschus cupreus*	Kashmir Musk Deer	Endangered	Decreasing	T
19	*Budorcas taxicolor*	Takin	Vulnerable	Decreasing	T
**20**	***Catagonus wagneri***	**Chacoan Peccary**	**Endangered**	**Decreasing**	**T**
21	*Sus cebifrons*	Visayan Warty Pig	Critically Endangered	Decreasing	T
**22**	***Inia geoffrensis***	**Boto**	**Data Deficient**	**Unknown**	**F**
23	*Saiga tatarica*	Mongolian Saiga	Critically Endangered	Decreasing	T
24	*Pantholops hodgsoni*	Chiru	Endangered	Decreasing	T
**25**	***Camelus bactrianus***	**Bactrian Camel**	**Critically Endangered**	**Decreasing**	**T**
**26**	***Boselaphus tragocamelus***	**Nilgai**	**Vulnerable**	**Stable**	**T**
27	*Tetracerus quadricornis*	Four-horned Antelope	Vulnerable	Decreasing	T
28	*Babyrousa babyrussa*	Hairy Babirusa	Vulnerable	Decreasing	T
29	*Babyrousa togeanensis*	Togian Islands Babirusa	Endangered	Decreasing	T
30	*Neophocaena phocaenoides*	Finless porpoise	Vulnerable	Decreasing	F,M

Species in bold are also among the top 30 most evolutionary distinct species (See Table 4). (T = Terrestrial, M = Marine, F = Freshwater).

**Table 2:** Top 30 conservation priority of carnivore species according with HEDGE analysis of the ‘pessimistic’ transformed data.

Rank	Species	Common name	IUCN Extinction Risk	IUCN Population Status	IUCN System
**1**	***Ailurus fulgens***	**Red Panda**	**Vulnerable**	**Decreasing**	**T**
**2**	***Ailuropoda melanoleuca***	**Giant Panda**	**Endangered**	**Decreasing**	**T**
**3**	***Monachus schauinslandi***	**Hawaiian Monk Seal**	**Critically endangered**	**Decreasing**	**T,M**
**4**	***Cynogale bennettii***	**Sunda Otter Civet**	**Endangered**	**Unknown**	**T,F**
5	*Pteronura brasiliensis*	Giant River Otter	Endangered	Decreasing	T,F
6	*Monachus monachus*	Mediterranean Monk Seal	Critically endangered	Decreasing	T,M
**7**	***Chrotogale owstoni***	**Owston's Civet**	**Vulnerable**	**Decreasing**	**T**
**8**	***Cryptoprocta ferox***	**Fossa**	**Vulnerable**	**Decreasing**	**T**
**9**	***Tremarctos ornatus***	**Spectacled Bear**	**Vulnerable**	**Decreasing**	**T**
**10**	***Liberiictis kuhni***	Liberian Mongoose	**Vulnerable**	**Decreasing**	**T**
11	*Enhydra lutris*	Sea Otter	Endangered	Stable	T,M
12	*Felis nigripes*	Black-footed cat	Vulnerable	Decreasing	T
13	*Macrogalidia musschenbroekii*	Sulawesi Palm Civet	Vulnerable	Decreasing	T
**14**	***Odobenus rosmarus***	**Walrus**	**Data Deficient**	**Unknown**	**T,M**
15	*Neofelis diardi*	Sunda Clouded Leopard	Vulnerable	Decreasing	T
16	*Lycaon pictus*	African Wild Dog	Endangered	Decreasing	T
17	*Arctictis binturong*	Binturong	Vulnerable	Decreasing	T
18	*Vormela peregusna*	European Marbled Polecat	Vulnerable	Decreasing	T
19	*Acinonyx jubatus*	Cheetah	Vulnerable	Decreasing	T
20	*Callorhinus ursinus*	Northern Fur Seal	Vulnerable	Decreasing	T,M
21	*Panthera uncia*	Snow Leopard	Endangered	Decreasing	T
22	*Amblonyx cinereus*	Asian Small-clawed Otter	Vulnerable	Decreasing	T,F,M
**23**	***Panthera onca***	**Jaguar**	**Not threatened**	**Decreasing**	**T**
24	*Ictailurus (Prionailurus) planiceps*	Flat-headed Cat	Endangered	Decreasing	T,F
25	*Urocyon littoralis*	Island Fox	Critically endangered	Decreasing	T
26	*Neofelis nebulosa*	Clouded Leopard	Vulnerable	Decreasing	T
27	*Fossa fossana*	Malagasy Civet	Not threatened	Decreasing	T
28	*Panthera tigris*	Tiger	Endangered	Decreasing	T
29	*Lutrogale perspicillata*	Smooth-coated Otter	Vulnerable	Unknown	T,F,M
30	*Leopardus jacobita*	Andean Cat	Endangered	Decreasing	T

Species in bold are also among the top 30 most evolutionary distinct species (See Table 5) (T = Terrestrial, M = Marine, F = Freshwater).

**Table 3:** Top cetartiodactylan and carnivore conservation priority species obtained from the multiple analysis and approaches.

Conservation Priorities	Common Name	IUCN Extinction Risk	IUCN Population Status	Raw-EDGE	Raw-HEDGE	Ult-EDGE	Ult-HEDGE	Overall Agreement
**CETARTIODACTYLA**								
***Lipotes vexillifer***	Baiji	Critically Endangered	Unknown	5	5	5	5	20
*Camelus bactrianus (ferus)*	Bactrian Camel	Critically Endangered	Decreasing	5	5	5	5	20
*Catagonus wagneri*	Chacoan Peccary	Endangered	Decreasing	5	5	5	5	20
*Pseudoryx nghetinhensis*	Saola	Critically Endangered	Decreasing	5	5	5	5	20
*Saiga tatarica*	Mongolian Saiga	Critically Endangered	Decreasing	5	5	5	5	20
*Tragulus nigricans*	Balabac Mouse Deer	Endangered	Decreasing	5	5	5	5	20
**CARNIVORA**								
*Ailuropoda melanoleuca*	Giant Panda	Endangered	decreasing	5	5	5	5	20
***Cynogale bennettii***	Sunda Otter Civet	Endangered	unknown	5	5	5	5	20
***Enhydra lutris***	Sea Otter	Endangered	stable	5	5	5	5	20
***Ictailurus (Prionailurus) planiceps***	Flat-headed Cat	Endangered	decreasing	5	5	5	5	20
*Leopardus jacobita*	Andean Cat	Endangered	decreasing	5	5	5	5	20
***Monachus monachus***	Mediterranean Monk Seal	Critically endangered	decreasing	5	5	5	5	20
***Monachus schauinslandi***	Hawaiian Monk Seal	Critically endangered	decreasing	5	5	5	5	20
*Panthera tigris*	Tiger	Endangered	Decreasing	5	5	5	5	20
***Pteronura brasiliensis***	Giant River Otter	Endangered	Decreasing	5	5	5	5	20
*Urocyon littoralis*	Island Fox	Critically endangered	Decreasing	5	5	5	5	20

In bold are aquatic and semi-aquatic species (see complete species list in Table S2).

The top-30 priority cetartiodactylan species for conservation according with the HEDGE/pessimistic metric are shown in Figure 1 and Table 1 (see Table S1 for more detailed results). Among the high-ranking conservation priorities are nearly all of the obligate and facultative freshwater species (Baiji, boto, Indus river, Ganges river dolphins, and finless porpoise), the semi-aquatic hippopotamus and pygmy hippopotamus, two marine species (sperm whale, Franciscana dolphin), and one species restricted to riverine habitats: the water chevrotain. The remaining species are terrestrial among them the Bactrian camel, Chacoan peccary, saola, Sambar deer, four-horned antelope, hairy Babirusa, Visayan warty pig, and several species of musk deer (Table 1).

The top 30 priority carnivore species for conservation are shown in Figure 2 and Table 2. The list includes four aquatic species (walrus, Hawaiian and Mediterranean monk seals, and the Northern fur seal), five semi-aquatic species (giant river otter, sea otter, European marbled polecat, Asian small clawed otter, and the smooth-coated otter), and one species restricted to riverine habitats (flat-headed cat). The remaining species are all terrestrial, where the red and giant panda ranked as the highest conservation priorities. Other high-ranking terrestrial species include eight species of cats: black-footed cat, Sunda clouded leopard, Cheetah, snow leopard, jaguar, clouded leopard, tiger, and the Andean mountain cat, Owston's and Sulawesi palm civets, Liberian Mongoose, fossa, spectacled bear, Malagasy civet, and the binturong (Table 2, Table S1).

### Congruence among methods

Differences among the myriad of available methodologies to estimate phylogeny-based conservation priorities remain unexplored. We explored 20 different combinations of analysis parameters, including the one used by Isaac et al. [31]. We find that method choice has marked impact on the exact ranking of conservation priorities. In fact, all 20 parameter combinations resulted in different species rankings, some individual species differing dramatically in rank from one to another (Tables 1,2,3,4,5, S1,S2). Nevertheless, if e.g. focusing on top priority species, such as top-30 lists based on each method, such lists largely overlap in the species contained despite differences in the exact rank of each species (Table 3, S2). For example, the Baiji dolphin was ranked as number one conservation priority by all 20 methods, and another 16 species were also listed among the top-30 conservation species by all methods, with slight variations in their relative ranking (Table S1, S2). Hence, overall congruence among methods when focusing on what species emerge as high priorities, rather than their exact rank, is relatively good.

**Table 4:** Top 30 most evolutionarily distinct (ED) species for Cetartiodactyla.

Rank	Species	Common name	IUCN Extinction Risk	IUCN Population Status	IUCN System	ED
**1**	***Lipotes vexillifer***	**Baiji**	**Critically Endangered**	**Extinct**	***F***	**0.70639556**
**2**	***Boselaphus tragocamelus***	**Nilgai**	**Vulnerable**	**Stable**	***T***	**0.67027222**
**3**	***Pontoporia blainvillei***	**Franciscana**	**Vulnerable**	**Decreasing**	***F***	**0.55766806**
**4**	***Inia geoffrensis***	**Boto**	**Data Deficient**	**Unknown**	***F***	**0.47185406**
**5**	***Physeter catodon***	**Sperm Whale**	**Vulnerable**	**Unknown**	***M***	**0.43151357**
**6**	***Hippopotamus amphibius***	**Hippopotamus**	**Vulnerable**	**Decreasing**	***T,F***	**0.38142334**
**7**	***Hexaprotodon (Choeropsis) liberiensis***	**Pygmy Hippopotamus**	**Endangered**	**Decreasing**	***T,F***	**0.35983234**
**8**	***Tragulus javanicus***	**Javan Chevrotain**	**Data Deficient**	**Unknown**	***T***	**0.32775791**
**9**	***Okapia johnstoni***	**Okapia**	**Near Threatened**	**Stable**	***T***	**0.32316998**
10	*Kogia simus*	Dwarf Sperm Whale	Data Deficient	Unknown	*M*	0.30603107
11	*Tragulus napu*	Greater Oriental Chevrotain	Least Concern	Decreasing	*T*	0.30280291
**12**	***Camelus dromedarius***	**Dromedary Camel**	**Data Deficient**	**Stable**	***T***	**0.28868987**
13	*Giraffa camelopardalis*	Giraffe	Least Concern	Decreasing	*T*	0.28773298
14	*Potamochoerus porcus*	Red River Hog	Least Concern	Decreasing	*T*	0.28629223
**15**	***Catagonus wagneri***	**Chacoan Peccary**	**Endangered**	**Decreasing**	***T***	**0.28045536**
**16**	***Cervus (Rusa) unicolor***	**Sambar**	**Vulnerable**	**Decreasing**	***T***	**0.27846745**
17	*Moschiola indica*	Indian Chevrotain	Least Concern	Unknown	*T*	0.27835511
18	*Moschiola kathygre*	Yellow-striped Chevrotain	Least Concern	Unknown	*T*	0.27835511
19	*Moschiola meminna*	White-spotted Chevrotain	Least Concern	Unknown	*T*	0.27835511
20	*Tragulus kanchill*	Lesser Oriental Chevrotain	Least Concern	Unknown	*T*	0.27620591
**21**	***Tragulus nigricans***	**Balabac Mouse Deer**	**Endangered**	**Decreasing**	***T***	**0.27620591**
**22**	***Tragulus versicolor***	**Silver-backed Chevrotain**	**Data Deficient**	**Decreasing**	***T***	**0.27620591**
23	*Hyperoodon ampullatus*	North Atlantic Bottlenose Whale	Data Deficient	Unknown	*M*	0.271927096
**24**	***Pseudoryx nghetinhensis***	**Saola**	**Critically Endangered**	**Decreasing**	***T***	**0.2645062**
25	*Kogia breviceps*	Pygmy Sperm Whale	Data Deficient	Unknown	*M*	0.26120107
**26**	***Camelus bactrianus***	**Bactrian Camel**	**Critically Endangered**	**Decreasing**	***T***	**0.25824787**
27	*Oreamnos americanus*	Mountain Goat	Least Concern	Stable	*T*	0.2571062
**28**	***Hyemoschus aquaticus***	**Water Chevrotain**	**Endangered**	**Decreasing**	**T**	**0.25301326**
**29**	***Tetracerus quadricornis***	**Four-horned Antelope**	**Vulnerable**	**Decreasing**	***T***	**0.24849422**
30	*Aepyceros melampus*	Impala	Least Concern	Stable	*T*	0.245038898

Species in bold are listed as conservation priorities by the multiple analysis (see Tables 3, S2). (T = Terrestrial, M = Marine, F = Freshwater).

**Table 5:** Top 30 most evolutionarily distinct (ED) species for Carnivora.

Rank	Species	Common name	IUCN Extinction Risk	IUCN Population Status	IUCN System	ED
**1**	***Odobenus rosmarus***	**Walrus**	**Data Deficient**	**Unknown**	***T,M***	**0.49564938**
**2**	***Ailurus fulgens***	**Red Panda**	**Vulnerable**	**Decreasing**	***T***	**0.42489602**
3	*Potos flavus*	Kinkajou	Least Concern	Decreasing	*T*	0.37237466
4	*Prionodon linsang*	Banded Linsang	Least Concern	Decreasing	*T*	0.35945443
5	*Genetta felina*	Common Genet	Least Concern	Stable	*T*	0.35655362
6	*Proteles cristatus*	Aardwolf	Least Concern	Stable	*T*	0.347611298
**7**	***Ailuropoda melanoleuca***	**Giant Panda**	**Endangered**	**Decreasing**	***T***	**0.33475031**
8	*Nyctereutes procyonoides*	Racoon Dog	Least Concern	Stable	*T*	0.308947596
**9**	***Cynogale bennettii***	**Sunda Otter Civet**	**Endangered**	**Unknown**	***T,F***	**0.29409765**
**10**	***Chrotogale owstoni***	**Owston's Civet**	**Vulnerable**	**Decreasing**	***T***	**0.285719397**
11	*Ictonyx libyca*	Libyan Striped Weasel	Least Concern	Unknown	*T*	0.27943796
**12**	***Pteronura brasiliensis***	**Giant River Otter**	**Endangered**	**Decreasing**	***T,F***	**0.27679501**
**13**	***Monachus schauinslandi***	**Hawaiian Monk Seal**	**Critically endangered**	**Decreasing**	***T,M***	**0.27469438**
14	*Arctogalidia trivirgata*	Small-toothed Palm Civet	Least Concern	Decreasing	*T*	0.27457579
15	*Suricata suricatta*	Meerkat	Least Concern	Unknown	*T*	0.26812055
16	*Nasua nasua*	South American Coati	Least Concern	Decreasing	*T*	0.26748003
17	*Taxidea taxus*	American Badger	Least Concern	Decreasing	*T*	0.25959407
18	*Fossa fossana*	Malagasy Civet	Not threatened	Decreasing	*T*	0.25833523
19	*Nandinia binotata*	African Palm Civet	Least Concern	Unknown	*T*	0.24800448
**20**	***Liberiictis kuhni***	**Liberian Mongoose**	**Vulnerable**	**Decreasing**	***T***	**0.246831998**
**21**	***Cryptoprocta ferox***	**Fossa**	**Vulnerable**	**Decreasing**	***T***	**0.24514323**
22	*Eira barbara*	Tayra	Least Concern	Decreasing	*T*	0.23918658
**23**	***Tremarctos ornatus***	**Spectacled Bear**	**Vulnerable**	**Decreasing**	***T***	**0.23783787**
**24**	***Felis nigripes***	**Black-footed cat**	**Vulnerable**	**Decreasing**	***T***	**0.23673007**
25	*Crocuta crocuta*	Spotted Hyaena	Least Concern	Decreasing	*T*	0.231299298
**26**	***Panthera onca***	**Jaguar**	**Not threatened**	**Decreasing**	***T***	**0.23093541**
**27**	***Galictis cuja***	**Lesser Grison**	**Least Concern**	**Unknown**	***T***	**0.22846741**
28	*Prionodon pardicolor*	Spotted Linsang	Least Concern	Unknown	*T*	0.22709743
29	*Bassariscus astutus*	Ringtail	Least Concern	Unknown	*T*	0.22423473
30	*Mellivora capensis*	Honey Badger	Least Concern	Decreasing	*T*	0.223801

Species in bold are listed as conservation priorities by the multiple analysis (see Tables 3, S2). (T = Terrestrial, M = Marine, F = Freshwater).

The species that rank high under a range of methods, in other words are high priority regardless of methodology (Table 3, S2), include most of the freshwater cetartiodactylans listed above, and other cetaceans such as blue whale, fin whale, sei whale, and the Vaquita. These also include the above mentioned aquatic carnivores as well as the Caspian seal, hooded seal, and the Galapagos, Australian, and Steller sea lions.

### Evolutionarily distinct species

The 30 most evolutionary distinct (ED) cetartiodactylans include three freshwater species (Baiji, Franciscana, and boto dolphins), two semi-aquatic species (hippopotamus and pygmy hippopotamus), five marine species (sperm whale, dwarf and pygmy sperm whales, North Atlantic bottlenose whale, and pygmy beaked whale), and 20 terrestrial species (Tables 4,5, S1). The Baiji is the most ED taxon in our analysis, and other high-ranking species include Nilgai, Franciscana, boto, sperm whale, dwarf sperm whale, the hippos, Java chevrotain, Greater mouse deer, okapi, Chacoan peccary, red river hog, and both Dromedary and Bactrian camels (Table 4,5, S1).

The most evolutionarily distinct carnivores include four aquatic species (walrus, Hawaiian monk seal, giant otter, and Sunda otter civet), and 26 terrestrial species among them the South American coati, red and giant panda, meerkat, fossa, tayra, kinkajou, jaguar, black-footed cat, spectacle bear, and several Civet species (see Table 4,5, S1). The walrus is the most ED taxon followed by the red panda, kinkajou, banded linsang, common genet and the aardwolf.

Evolutionarily distinct species are disproportionally on the decline and more poorly known than the average species in these two orders, and relatively few high ED species are stable, and none is on the increase (χ^2^ = 15.8, p = 0.0012, df = 3).

## Discussion

Here we provide phylogenetic conservation priorities for the two largest groups of aquatic mammals and their terrestrial relatives (Cetartiodactyla and Carnivora), based on phylogenetic information and species imperilment. Our results provide a more detailed phylogenetic conservation resource for these two groups than prior work, and guideline for allocation of future conservation effort (Figure 1, Tables 1,2,3,4,5, S1,S2).

Our findings indicate that evolutionarily distinctiveness and conservation priorities are in general distributed among terrestrial and aquatic species in proportion to their diversity (Tables 1,2,3,4,5, S1,S2). However, several observations highlight the need for special conservation effort for aquatic mammals. Many aquatic mammals are evolutionarily distinct species adapted to fragile ecosystems where their populations have suffered high levels of human exploitation. For instance, seven of the extant obligated and facultative freshwater cetartiodactylan species (Baiji, Boto, Ganges and Indus River Dolphins, Finless Porpoise, hippo and pygmy hippo) rank as top conservation priorities. Most of these can be characterized as relict species-poor lineages, that have diversified little or not at all, following transition to freshwater [44], [45]. We note that freshwater populations of the Irrawaddy dolphin (*Orcaella brevirostris*) are also critically endangered [see 19], but as marine populations are doing relatively better the species does not emerge as particularly high priority using the current methodology. However, this also demonstrates high relative threats to freshwater mammal species and populations. The high conservation priority of freshwater mammals may relate to various factors. Habitat pressure is particularly high in freshwater systems where some rivers have become highly polluted, both chemically and acoustically. In addition, many of the main freshwater streams are dammed and suffer from heavy boat traffic posing a direct threat to the animals. For example, the Baiji (Yangtze) river dolphin is the highest-ranking conservation priority of all species considered in this study (Table S1). Although it is currently characterized as critically endangered with unknown population status, it is thought to have recently gone extinct, due to a combination of factors with the most important probably being incidental by-catch using rolling hooks, nets, and electro-fishing, but also other factors such as noise pollution, and direct impact with boats [46], [47]. Another high-ranking conservation priority inhabitant of the Yangtze River is the finless porpoise, which scientists fear may be facing a similar fate as the Baiji [48]. Furthermore, the highly evolutionarily distinct walrus and sperm whale also are relict species. Both species have suffered intense historical hunting, and currently there is insufficient knowledge of their population trend. Although protected by law these marine species are also threatened by climate change, a concern that may require new management approaches [49], [50].

Among the terrestrial species that rank among the top conservation priorities, between 60–70% have highly restricted ranges where they are mainly threatened by habitat loss and harvesting [1]. For instance, the red panda populations are mainly affected by habitat fragmentation and poaching [51]–[53] which is causing population bottlenecks and inbreeding [54]. Similarly, the giant panda and the white-spotted Chevrotain may be facing local extinction across their distribution due to intense habitat fragmentation [55], [56].

The majority of the top 60 conservation priority species are under some kind of law protection (e.g., CITES, hunting regulations) and occur in one or more protected areas [see 19]. Some species like the tiger and giant panda have been the focus of intense public and conservation attention. Nevertheless, despite their occurrence in protected areas, and other existing conservation efforts, these species are nearly universally declining [1], [2], [19]. Overall, of the 620 species considered in the analysis, populations are decreasing for 46% of the species, 24% are stable or increasing, and for 30% population trends are unknown. For the 60 top conservation priority species, populations of 87% are decreasing, and for another 10% data are insufficient to tell. Therefore, strikingly, only 3% of the top conservation priority species are thought to be stable or on the increase. Furthermore, it is interesting to observe that more highly evolutionarily distinct species are declining and unknown, and fewer are stable and on the increase (zero) than expected if they represented a random draw of species from these two orders. Hence there may be something about high ED species that makes them more vulnerable to human activities, while these are arguably particularly important to conserve. Thus, our findings leave no doubt that for those species that are, or arguably should be, receiving the greatest conservation effort, including large charismatic mammals that are conservation icons, our current effort seems to be insufficient to maintain population sizes [19], [57]–[59]. As exemplified by the red panda, reduced population sizes can only lead to increased risk of extinction, both through direct constraints, and further problems such as reduced genetic variability, and lack of populations to boost variability in depleted populations [53]. Why is our effort failing? The main causes of population decrease are in most cases some kind of extraction. Overall, populations of nearly 80% of the species are thought to be decreasing due to hunting, incidental mortality or illegal trading, and close to 60% are on the decrease due to habitat loss, which are general threats affecting most mammals. In this light of conservation effort that currently seems not to be achieving its goals, analyses such as the present are important. Clearly, there is an urgent need to focus conservation effort so that at least some of the species that are deemed to be relatively important are secured into the future, and it is important to understand why protected species are still on the decline, and why high ED species are faring more poorly than lower ED species.

We note that we here consider only extinction risk and evolutionarily distinctiveness. Many other factors contribute to conservation decision-making. These include ecological function and importance of species, economic value, and charisma among others. Perhaps, in light of ongoing population declines in the vast majority of top conservation priority cetartiodactylans and carnivores, one of the first and most important factors to consider is feasibility of successful conservation strategies [1], [2]. However, measures such as the one we provide here may help to focus attention on species whose loss would prune disproportionably deep branches of the tree of life.

## Supporting Information

Table S1 **Full data set for evolutionary distinctiveness, EDGE and HEDGE calculations using TUATARA (Carnivores = 387, Cetartiodactyls = 333).**
(XLS)

Table S2 **Consensus list of conservation priority species obtained from the multiple analysis and approaches.** In bold are aquatic and semi-aquatic species.
(DOC)

## References

[1] Schipper J, Chanson JS, Chiozza F, Cox NA, Hoffmann M (2008). The status of the World's land and marine mammals: diversity, threat, and knowledge.. Science.

[2] Williams N (2009). Red list species update fears.. Current Biology.

[3] Dirzo R, Mendoza E, Ortiz P (2007). Size-related differential seed predation in a heavily defaunated neotropical rain forest.. Biotropica.

[4] Kokko H, Lindstrom J, Ranta E (1997). Risk analysis of hunting of seal populations in the Baltic.. Conservation Biology.

[5] Clapham PJ (1999). Baleen whales: conservation issues and the status of the most endangerous populations.. Mammal Review.

[6] Clapham PJ, Aguilar A, Hatch LT (2008). Determining spatial and temporal scales for management: lessons from whaling.. Marine Mammal Science.

[7] Springer AM, Estes JA, van Vliet GB, Williams TM, Doak DF (2008). Mammal-eating killer whales, industrial whaling, and the sequential megafaunal collapse in the North Pacific Ocean: A reply to critics of Spring et al. 2003.. Marine Mammal Science.

[8] Lea MA, Guinet C, Cherel Y, Duhamel G, Dubroca L (2006). Impacts of climate anomalies on provisioning strategies of a southern ocean predator.. Marine Ecology Progress Series.

[9] Simmonds MP, Isaac SJ (2007). The impacts of climate change on marine mammals: early signs of significant problems.. Oryx.

[10] Kirkwood R, Hume F, Hindell M (2008). Sea temperature variations mediate annual changes in the diet of Australian fur seals in Bass Strait.. Marine Ecology Progress Series.

[11] Higdon JW, Ferguson SH (2009). Loss of Arctic sea ice causing punctuated change in sightings of killer whales (*Orcinus orca*) over the past century.. Ecological Applications.

[12] Regehr EV, Hunter CM, Caswell H, Amstrup SC, Stirling I (2010). Survival and breeding of polar bears in the southern Beaufort Sea in relation to sea ice.. Journal of Animal Ecology.

[13] Morizur Y, Berrow SD, Tregenza NJC, Couperus AS, Pouvreau S (1999). Incidental catches of marine-mammals in pelagic trawler fisheries of the northeast Atlantic.. Fisheries Research.

[14] Gerrodette T, Forcada J (2005). Non-recovery of two spotted and spinner dolphin populations in the eastern tropical Pacific Ocean.. Marine Ecology Progress Series.

[15] Wade PR, Watters GM, Gerrodette T, Reilly SB (2007). Depletion of spotted and spinner dolphins in the eastern tropical Pacific: modeling hypotheses for their lack of recovery.. Marine Ecology Progress Series.

[16] Cramer KL, Perryman WL, Gerrodette T (2008). Declines in reproductive output in two dolphin populations depleted by the yellowfin tuna purse-seine fishery.. Marine Ecology Progress Series.

[17] Hamer DJ, Ward TM, McGarvey R (2008). Measurement, management, and mitigation of operational interactions between the South Australian sardine fishery and short-beaked common dolphins (*Delphinus delphis*).. Biological Conservation.

[18] Plaganyi EE, Butterworth DS (2008). Competition between marine mammals and fisheries- Can we successfully model this using ECOPATH with ECOSIM?. Reconciling Fisheries with Conservation.

[19] International Union for Conservation of Nature (IUCN) Red List of Threatened Species.. http://www.iucnredlist.org.

[20] Zydelis R, Wallace BP, Gilman EL, Werner TB (2009). Conservation of marine megafauna through minimization of fisheries bycatch.. Conservation Biology.

[21] Cardillo M, Mace GM, Gittleman JL, Purvis A (2006). Latent extinction risk and the future battlegrounds of mammal conservation.. Proceedings of the National Academy of Sciences of the United States of America.

[22] Faith DP (1992). Conservation evaluation and phylogenetic diversity.. Biological Conservation.

[23] Faith DP (2002). Quantifying biodiversity: a phylogenetic perspective.. Conservation Biology.

[24] Faith DP, Reid CAM, Hunter J (2004). Integrating phylogenetic diversity, complementarity, and endemism for conservation assessment.. Conservation Biology.

[25] Redding DW, Mooers AØ (2006). Incorporating evolutionary measures into conservation prioritization.. Conservation Biology.

[26] Steel M, Mimoto A, Mooers AØ (2007). Hedging one's bets: quantifying a taxon's expected contribution to future phylogenetic diversity.. Evolutionary Bioinformatics.

[27] Mooers AØ, Faith DP, Maddison WP (2008). Converting endangered species categories to probabilities of extinction for phylogenetic conservation prioritization.. PLoS One.

[28] Redding DW, Hartmann K, Mimoto A, Bokal D, Devos M (2008). Evolutionarily distinctive species often capture more phylogenetic diversity than expected.. Journal of Theoretical Biology.

[29] Beenaerts N, Pethiyagoda R, Ng PKL, Yeo DC, Bex GJ (2010). Phylogenetic diversity of Sri Lankan freshwater crabs and its implications for conservation.. Molecular Ecology.

[30] Vane-Wright RI, Humphries CJ, Williams PH (1991). What to protect—systematics and the agony of choice.. Biological Conservation.

[31] Isaac NJB, Turvey ST, Collen B, Waterman C, Baillie JEM (2007). Mammals on the EDGE: conservation priorities based on threat and phylogeny.. PLoS ONE.

[32] Evolutionarily Distinct & Globally Endangered.. http://www.edgeofexistence.org/.

[33] Russell GJ, Brooks TM, McKinney MM, Anderson CG (1998). Present and future taxonomic selectivity in bird and mammal extinctions.. Conservation Biology.

[34] Davies JT, Fritz SA, Grenyer R, Orme CDL, Bielby J (2008). Phylogenetic tress and the future of mammalian biodiversity.. Proceedings of the National Academy of Sciences of the United States of America.

[35] Agnarsson I, May-Collado LJ (2008). The phylogeny of Cetartiodactyla: the importance of dense taxon sampling, missing data, and the remarkable promise of Cytochrome b to provide reliable species-level phylogenies.. Molecular Evolution and Phylogenetics.

[36] Agnarsson I, Kuntner M, May-Collado LJ (2010). Dogs, cats, and kin: A molecular species-level phylogeny of Carnivora.. Molecular Phylogenetics and Evolution.

[37] YouTheria.. http://www.utheria.org/.

[38] Bininda-Emonds O, Cardillo M, Jones KE, MacPhee RDE, Beck RMD (2007). The delayed rise of present-day mammals.. Nature.

[39] Purvis A, Gittleman JL, Cowlishaw G, Mace GM (2000). Predicting extinction risk in declining species.. Proceedings of the Royal Society B: Biological Sciences.

[40] Faith DP (2008). Threatened species and the potential loss of phylogenetic diversity: conservation scenarios based on estimated extinction probabilities and phylogenetic risk analysis.. Conservation Biology.

[41] Kuntner M, May-Collado LJ, Agnarsson I (2011). Phylogeny and conservation priorities of afrotherian mammals (Afrotheria, Mammalia).. Zoologica Scripta.

[42] Maddison WP, Mooers AØ (2007). Tuatara: Conservation priority in a phylogenetic context, Version 1.0.. http://mesquiteproject.org/packages/tuatara.

[43] Maddison WP, Maddison DR (2009). Mesquite: a modular system for evolutionary analysis.. http://mesquiteproject.org.

[44] Hamilton H, Caballero S, Collins AG, Brownell RL (2001). Evolution of river dolphins.. Proceedings of the Royal Society of London.

[45] Boisserie JR, Lihoreau F, Brunet M (2005). The position of Hippopotamidae within Cetartiodactyla.. Proceedings of the National Academy of Sciences of the United States of America.

[46] Pyeson ND (2009). Requiem for *Lipotes*: an evolutionary perspective on marine mammal extinction.. Marine Mammal Science.

[47] Turvey ST, Pitman RL, Taylor BL, Barlow J, Akamatsu T (2007). First human-caused extinction of a cetacean species?. Biology Letters.

[48] Wang D (2009). Population status, threats and conservation of the Yantgze finless porpoise.. Chinese Science Bulletin.

[49] Robards MD, Burns JJ, Meek CL, Watson A (2009). Limitations of an optimum sustainable population or potential biological removal approach for conserving marine mammals: Pacific walrus case study.. Journal of Environmental Management.

[50] Whitehead H, Rendell L, Osborne RW, Wursig B (2004). Culture and conservation of non-humans with reference to whales and dolphins: review and new directions.. Biological Conservation.

[51] Yonzon PB, Hunter ML (1991). Conservation of the red panda *Aiulurus fulgens*.. Biological Conservation.

[52] Fox J, Yonzon P, Podger N (1996). Mapping conflicts between biodiversity and human needs in Langtang National Park, Nepal.. Conservation Biology.

[53] Choudhury A (2001). An overview of the status and conservation of the red panda *Ailurus fulgens* in India, with reference to its global status.. Oryx.

[54] Li M, Wei F, Goossens B, Feng Z, Hidetoshi BT (2005). Mitochondrial phylogeography and subspecific variation in the red panda (*Aiulurus fulgens*): implications for conservation.. Molecular Phylogenetics and Evolution.

[55] Wang XZ, Xu WH, Ouyang ZY (2009). Integrating population size analysis into habitat suitability assessment: implications for giant panda conservation in the Minshan Mountains, China.. Ecological Research.

[56] Ran JH, Du BB, Yue BS (2009). Conservation of the endangered giant panda *Ailuropoda melanoleuca* in China: successes and challenges.. Oryx.

[57] Brodie JF (2009). Is research effort allocated efficiently for conservation? Felidae as a global case study.. Biodiversity and Conservation.

[58] Harihar A, Prasad DL, Ri C, Pandav B, Goyal SP (2009). Losing ground: tiger *Panthera tigris* in the norh-western Shivalik landscape of India.. Oryx.

[59] Sanchez-Mercado A, Ferrer-Paris JR, Yerena E, Garcia-Rangel S, Rodriguez-Clark M (2008). Factors affecting poaching risk to vulnerable Andean bears *Tremarctos ornatus* in the Cordillera de Merida, Venezuela: space, parks, and people.. Oryx.

