# Supplementary material for: Phylogenetic Analysis of Conservation Priorities for Aquatic Mammals and Their Terrestrial Relatives, with a Comparison of Methods
Source: PLoS One. 2011 Jul 25;6(7):e22562. doi: 10.1371/journal.pone.0022562 (PMC3143159; doi:10.1371/journal.pone.0022562)
Supplement: Table S2 — Consensus list of conservation priority species obtained from the multiple analysis and approaches. In bold are aquatic and semi-aquatic species. (DOC) [file pone.0022562.s002.doc]

Table S2. Consensus list of conservation priority species obtained from the multiple analysis and approaches. In bold are aquatic and semi-aquatic species.

| **Conservation Priorities** | **Common Name** | **IUCN Extinction Risk** | **IUCN Population Status** | **Raw-EDGE** | **Raw-HEDGE** | **Ult-EDGE** | **Ult-HEDGE** | **Overall Agreement** |
| --- | --- | --- | --- | --- | --- | --- | --- | --- |
| **Cetartiodactyla** | | | | | | | | |
| ***Lipotes vexillifer*** | Baiji | Critically Endangered | Unknown | 5 | 5 | 5 | 5 | 20 |
| *Camelus bactrianus (ferus)* | Bactrian Camel | Critically Endangered | Decreasing | 5 | 5 | 5 | 5 | 20 |
| *Catagonus wagneri* | Chacoan Peccary | Endangered | Decreasing | 5 | 5 | 5 | 5 | 20 |
| *Pseudoryx nghetinhensis* | Saola | Critically Endangered | Decreasing | 5 | 5 | 5 | 5 | 20 |
| *Saiga tatarica* | Mongolian Saiga | Critically Endangered | Decreasing | 5 | 5 | 5 | 5 | 20 |
| *Tragulus nigricans* | Balabac Mouse Deer | Endangered | Decreasing | 5 | 5 | 5 | 5 | 20 |
| ***Hexaprotodon (Choeropsis) liberiensis*** | Pygmy Hippopotamus | Endangered | Decreasing | 5 | 5 | 5 | 4 | 19 |
| *Hyemoschus aquaticus* | Water Chevrotain | Endangered | Decreasing | 5 | 5 | 4 | 5 | 19 |
| ***Platanista gangetica*** | Ganges River Dolphin | Endangered | Decreasing | 5 | 5 | 5 | 4 | 19 |
| ***Platanista minor*** | Indus River Dolphin | Endangered | Decreasing | 5 | 5 | 5 | 4 | 19 |
| *Pantholops hodgsonii* | Chiru | Endangered | Decreasing | 5 | 5 | 4 | 4 | 18 |
| ***Balaenoptera musculus*** | Blue Whale | Endangered | Increasing | 5 | 3 | 5 | 4 | 17 |
| *Beatragus hunteri* | Hirola | Critically Endangered | Decreasing | 3 | 3 | 5 | 5 | 16 |
| *Elaphurus davidianus* | Père David’s Deer | Critically Endangered | extinct in the wild | 3 | 3 | 5 | 5 | 16 |
| *Babyrousa togeanensis* | Togian Islands Babirusa | Endangered | decreasing | 4 | 1 | 5 | 5 | 15 |
| *Sus cebifrons* | Visayan Warty Pig | Critically Endangered | Decreasing | 5 | 5 | 3 | 2 | 15 |
| *Axis kuhlii* | Bawean Deer | Critically Endangered | Decreasing | 3 | 4 | 3 | 3 | 13 |
| *Bubalus mindorensis* | Mindoro Dwarf Buffalo | Critically Endangered | Decreasing | 3 | 3 | 3 | 4 | 13 |
| *Cephalophus adersi* | Aders’ Duiker | Critically Endangered | Decreasing | 3 | 3 | 4 | 3 | 13 |
| *Cervus (Rucervus) eldii* | Eld’s Deer | Endangered | Decreasing | 3 | 0 | 5 | 5 | 13 |
| *Porcula salvania* | Pygmy Hog | Critically Endangered | Decreasing | 1 | 3 | 5 | 4 | 13 |
| *Procapra przewalskii* | Przewalski's Gazelle | Endangered | Decreasing | 4 | 2 | 4 | 2 | 12 |
| *Addax nasomaculatus* | Addax | Critically Endangered | Decreasing | 2 | 2 | 4 | 3 | 11 |
| ***Phocoena sinus*** | Vaquita | Critically Endangered | Decreasing | 4 | 2 | 3 | 2 | 11 |
| ***Physeter catodon*** | Sperm Whale | Vulnerable | Unknown | 3 | 3 | 3 | 2 | 11 |
| *Gazella (Nanger) dama* | Dama Gazelle | Critically Endangered | Decreasing | 4 | 3 | 3 | 0 | 10 |
| *Hemitragus (Arabitragus) jayakari* | Arabian Tahr | Endangered | Decreasing | 0 | 0 | 5 | 5 | 10 |
| ***Hippopotamus amphibius*** | Hippopotamus | Vulnerable | Decreasing | 3 | 4 | 1 | 2 | 10 |
| *Sus oliveri* | Oliver's Warty Pig | Endangered | Decreasing | 0 | 0 | 5 | 4 | 9 |
| *Axis calamianensis* | Calamian Hog Deer | Endangered | Decreasing | 1 | 3 | 1 | 3 | 8 |
| ***Balaenoptera physalus*** | Fin Whale | Endangered | Unknown | 0 | 0 | 5 | 3 | 8 |
| *Bos sauveli* | Grey Ox | Critically Endangered | Unknown | 4 | 3 | 1 | 0 | 8 |
| *Cervus (Rusa) unicolor* | Sambar | Vulnerable | Decreasing | 3 | 3 | 1 | 1 | 8 |
| ***Pontoporia blainvillei*** | Franciscana | Vulnerable | Decreasing | 3 | 3 | 1 | 1 | 8 |
| *Dama mesopotamica* | Persian Fallow Deer | Endangered | Increasing | 4 | 3 | 0 | 0 | 7 |
| *Cephalophus jentinki* | Jentink’s Duiker | Endangered | Decreasing | 3 | 3 | 0 | 0 | 6 |
| *Hemitragus hylocrius* | Nilgiri tahr | Endangered | Decreasing | 0 | 2 | 2 | 2 | 6 |
| *Rusa alfredi* | Phillipine Spotted Deer) | Endangered | Decreasing | 0 | 1 | 2 | 3 | 6 |
| *Axis porcinus* | Hog Deer | Endangered | Decreasing | 0 | 3 | 0 | 2 | 5 |
| *Babyrousa babyrussa* | Hairy Babirusa | Vulnerable | Decreasing | 1 | 1 | 1 | 2 | 5 |
| *Ammodorcas clarkei* | Dibatag | Vulnerable | Decreasing | 1 | 1 | 1 | 1 | 4 |
| *Bubalus bubalis* | Water Buffalo | Endangered | Decreasing | 0 | 2 | 0 | 2 | 4 |
| *Bubalus depressicornis* | Lowland Anoa | Endangered | Decreasing | 0 | 2 | 0 | 2 | 4 |
| *Bubalus quarlesi* | Mountain Anoa | Endangered | Decreasing | 0 | 1 | 1 | 2 | 4 |
| *Kobus megaceros* | Nile Lechwe | Endangered | Decreasing | 3 | 0 | 1 | 0 | 4 |
| *Moschus anhuiensis* | Anhui Musk Deer | Endangered | Decreasing | 0 | 2 | 0 | 2 | 4 |
| *Moschus berezovskii* | Forest Musk Deer | Endangered | Decreasing | 0 | 2 | 0 | 2 | 4 |
| *Moschus chrysogaster* | Alpine Musk Deer | Endangered | Decreasing | 0 | 2 | 0 | 2 | 4 |
| *Moschus cupreus* | Kashmir Musk Deer | Endangered | Decreasing | 0 | 2 | 0 | 2 | 4 |
| *Moschus fuscus* | Black Musk Deer | Endangered | Decreasing | 0 | 2 | 0 | 2 | 4 |
| *Moschus leucogaster* | Himalayan Musk Deer | Endangered | Decreasing | 0 | 2 | 0 | 2 | 4 |
| *Moschus moschiferus* | Siberian Musk Deer | Vulnerable | Decreasing | 1 | 2 | 0 | 1 | 4 |
| *Oryx dammah* | Scimitar horned Oryx | Critically Endangered | extinct in the wild | 2 | 0 | 2 | 0 | 4 |
| *Ammotragus lervia* | Aoudad | Vulnerable | Decreasing | 1 | 0 | 1 | 1 | 3 |
| *Budorcas taxicolor* | Takin | Vulnerable | Decreasing | 1 | 2 | 0 | 0 | 3 |
| *Hippocamelus bisulcus* | Patagonian Huemul | Endangered | Decreasing | 0 | 3 | 0 | 0 | 3 |
| *Tetracerus quadricornis* | Four-horned Antelope | Vulnerable | Decreasing | 1 | 1 | 1 | 0 | 3 |
| *Babyrousa celebensis* | Sulawesi Babirusa | Vulnerable | decreasing | 0 | 0 | 1 | 1 | 2 |
| ***Balaenoptera borealis*** | Sei Whale | Endangered | unknown | 0 | 0 | 2 | 0 | 2 |
| *Bos javanicus* | Banteng | Endangered | decreasing | 2 | 0 | 0 | 0 | 2 |
| *Boselaphus tragocamelus* | Nilgai | Vulnerable | stable | 1 | 1 | 0 | 0 | 2 |
| *Dorcatragus megalotis* | Beira | Vulnerable | decreasing | 1 | 0 | 1 | 0 | 2 |
| *Hydropotes inermis* | Chinese Water Deer | Vulnerable | decreasing | 1 | 0 | 1 | 0 | 2 |
| ***Inia geoffrensis*** | Boto | Data Deficient | unknown | 1 | 1 | 0 | 0 | 2 |
| ***Neophocaena phocaenoides*** | Finless Porpoise | Vulnerable | decreasing | 1 | 1 | 0 | 0 | 2 |
| *Pseudois schaeferi* | Dwarf Bharal | Endangered | decreasing | 2 | 0 | 0 | 0 | 2 |
| *Tragelaphus buxtoni* | Mountain Nyala | Endangered | decreasing | 2 | 0 | 0 | 0 | 2 |
| *Cervus duvaucelii* | Barasingha | Vulnerable | decreasing | 1 | 0 | 0 | 0 | 1 |
| *Okapia johnstoni* | Okapia | Near Threatened | stable | 1 | 0 | 0 | 0 | 1 |
| **Carnivora** | | | | | | | | |
| *Ailuropoda melanoleuca* | Giant Panda | Endangered | decreasing | 5 | 5 | 5 | 5 | 20 |
| ***Cynogale bennettii*** | Sunda Otter Civet | Endangered | unknown | 5 | 5 | 5 | 5 | 20 |
| ***Enhydra lutris*** | Sea Otter | Endangered | stable | 5 | 5 | 5 | 5 | 20 |
| ***Ictailurus (Prionailurus) planiceps*** | Flat-headed Cat | Endangered | decreasing | 5 | 5 | 5 | 5 | 20 |
| *Leopardus jacobita* | Andean Cat | Endangered | decreasing | 5 | 5 | 5 | 5 | 20 |
| ***Monachus monachus*** | Mediterranean Monk Seal | Critically endangered | decreasing | 5 | 5 | 5 | 5 | 20 |
| ***Monachus schauinslandi*** | Hawaiian Monk Seal | Critically endangered | decreasing | 5 | 5 | 5 | 5 | 20 |
| *Panthera tigris* | Tiger | Endangered | Decreasing | 5 | 5 | 5 | 5 | 20 |
| ***Pteronura brasiliensis*** | Giant River Otter | Endangered | Decreasing | 5 | 5 | 5 | 5 | 20 |
| *Urocyon littoralis* | Island Fox | Critically endangered | Decreasing | 5 | 5 | 5 | 5 | 20 |
| *Lycaon pictus* | African Wild Dog | Endangered | Decreasing | 5 | 5 | 5 | 4 | 19 |
| *Ailurus fulgens* | Red Panda | Vulnerable | Decreasing | 4 | 5 | 4 | 5 | 18 |
| *Panthera uncia* | Snow Leopard | Endangered | Decreasing | 5 | 5 | 4 | 4 | 18 |
| *Cryptoprocta ferox* | Fossa | Vulnerable | Decreasing | 3 | 5 | 3 | 5 | 16 |
| *Cuon alpinus* | Dhole | Endangered | Decreasing | 2 | 4 | 5 | 5 | 16 |
| *Macrogalidia musschenbroekii* | Sulawesi Palm Civet | Vulnerable | Decreasing | 3 | 5 | 3 | 5 | 16 |
| ***Prionailurus viverrinus*** | Fishing Cat | Endangered | Decreasing | 4 | 4 | 4 | 4 | 16 |
| *Tremarctos ornatus* | Spectacled Bear | Vulnerable | Decreasing | 3 | 5 | 3 | 5 | 16 |
| *Lynx pardinus* | Iberian Lynx | Critically endangered | Decreasing | 4 | 1 | 5 | 5 | 15 |
| ***Neophoca cinerea*** | Australian Sea Lion | Endangered | Decreasing | 4 | 4 | 3 | 4 | 15 |
| *Procyon pygmaeus* | Pygmy Raccoon | Critically endangered | Decreasing | 5 | 4 | 4 | 2 | 15 |
| ***Lutra sumatrana*** | Hairy-nosed Otter | Endangered | Decreasing | 4 | 3 | 4 | 3 | 14 |
| ***Eumetopias jubatus*** | Steller Sea Lion | Endangered | Decreasing | 3 | 2 | 4 | 4 | 13 |
| *Canis himalayensis* | Himalayan Wolf | Critically endangered | Unknown | 3 | 3 | 3 | 3 | 12 |
| *Galidictis grandidieri* | Giant striped Mongoose | Endangered | Decreasing | 5 | 2 | 4 | 1 | 12 |
| ***Lontra felina*** | Marine Otter | Endangered | Decreasing | 4 | 3 | 2 | 2 | 11 |
| *Vormela peregusna* | European Marbled Polecat | Vulnerable | decreasing | 3 | 3 | 3 | 2 | 11 |
| *Arctictis binturong* | Binturong | Vulnerable | Decreasing | 1 | 3 | 3 | 3 | 10 |
| *Chrotogale owstoni* | Owston’s Civet | Vulnerable | Decreasing | 3 | 5 | 1 | 0 | 9 |
| *Acinonyx jubatus* | Cheetah | Vulnerable | Decreasing | 1 | 3 | 1 | 3 | 8 |
| ***Callorhinus ursinus*** | Northern Fur Seal | Vulnerable | Decreasing | 1 | 3 | 1 | 3 | 8 |
| *Canis simensis* | Ethiopian Wolf | Endangered | Decreasing | 0 | 0 | 4 | 4 | 8 |
| *Liberiictis kuhni* | Liberian Mongoose | Vulnerable | Decreasing | 3 | 5 | 0 | 0 | 8 |
| *Viverra civettina* | Malabar Civet | Critically endangered | Unknown | 4 | 1 | 1 | 2 | 8 |
| ***Lontra provocax*** | Southern River Otter | Endangered | Decreasing | 3 | 0 | 2 | 2 | 7 |
| *Melursus ursinus* | Sloth Bear | Vulnerable | Decreasing | 0 | 0 | 3 | 4 | 7 |
| ***Phoca caspica*** | Caspian Seal | Endangered | Decreasing | 2 | 0 | 3 | 2 | 7 |
| *Canis rufus* | Red Wolf | Critically endangered | Increasing | 3 | 0 | 3 | 0 | 6 |
| *Felis nigripes* | Black-footed cat | Vulnerable | Decreasing | 3 | 3 | 0 | 0 | 6 |
| *Helarctos malayanus* | Malayan Sun Bear | Vulnerable | Decreasing | 0 | 0 | 3 | 3 | 6 |
| *Catopuma badia* | Borneo Bay Cat | Endangered | Decreasing | 2 | 0 | 2 | 1 | 5 |
| ***Zalophus wollebaeki*** | Galápagos Sea Lion | Endangered | Decreasing | 3 | 0 | 2 | 0 | 5 |
| ***Cystophora cristata*** | Hooded Seal | Vulnerable | Decreasing | 0 | 0 | 1 | 3 | 4 |
| *Leopardus tigrinus* | Oncilla | Vulnerable | Decreasing | 0 | 0 | 1 | 3 | 4 |
| *Neofelis diardi* | Sunda Clouded Leopard | Vulnerable | Decreasing | 1 | 2 | 0 | 1 | 4 |
| *Fossa fossana* | Malagasy Civet | Not threatened | Decreasing | 1 | 2 | 0 | 0 | 3 |
| ***Odobenus rosmarus*** | Walrus | Data Deficient | Unknown | 1 | 2 | 0 | 0 | 3 |
| ***Amblonyx (Aonyx) cinereus (cinerea)*** | Asian Small-clawed Otter | Vulnerable | Decreasing | 1 | 1 | 0 | 0 | 2 |
| ***Arctocephalus galapagoensis*** | Galápagos Fur Seal | Endangered | Decreasing | 2 | 0 | 0 | 0 | 2 |
| *Mustela nigripes* | Black footed Ferret | Endangered | Increasing | 1 | 1 | 0 | 0 | 2 |
| *Neofelis nebulosa* | Clouded Leopard | Vulnerable | Decreasing | 0 | 1 | 0 | 1 | 2 |
| *Panthera leo* | Lion | Vulnerable | Decreasing | 0 | 0 | 1 | 1 | 2 |
| *Canis indica* | Indian Wolf | Endangered | Stable | 0 | 0 | 1 | 0 | 1 |
| *Galictis cuja* | Lesser Grison | Least Concern | Unknown | 0 | 1 | 0 | 0 | 1 |
| *Helogale parvula* | Common Dwarf Mongoose | Least Concern | Stable | 0 | 1 | 0 | 0 | 1 |
| *Herpestes urva* | Crab eating Mongoose | Least Concern | Stable | 0 | 1 | 0 | 0 | 1 |
| ***Lutrogale perspicillata*** | Smooth-coated Otter | Vulnerable | Unknown | 0 | 1 | 0 | 0 | 1 |
| *Oncifelis guigna* | Guina | Vulnerable | Decreasing | 0 | 0 | 0 | 1 | 1 |
| *Panthera onca* | Jaguar | Not threatened | Decreasing | 0 | 1 | 0 | 0 | 1 |
| *Paradoxurus zeylonensis* | Golden Palm Civet | Vulnerable | Decreasing | 1 | 0 | 0 | 0 | 1 |
| ***Ursus maritimus*** | Polar Bear | Vulnerable | Decreasing | 0 | 0 | 1 | 0 | 1 |
